# Extraction of Cellulose-Based Polymers from Textile Wastes

**DOI:** 10.3390/polym14102063

**Published:** 2022-05-18

**Authors:** Helena P. Felgueiras, Jorge Padrão, Joana C. Antunes

**Affiliations:** Centre for Textile Science and Technology (2C2T), University of Minho, Campus de Azurém, 4800-058 Guimarães, Portugal; padraoj@2c2t.uminho.pt (J.P.); joana.antunes@2c2t.uminho.pt (J.C.A.)

The extraction and exploration of cellulose-based polymers is an exciting area of research. For many years, wood (especially from bleached kraft wood pulp) was considered the main source of cellulosic compounds because of its abundance in nature [1,2]. However, in the past decade, researchers have been devoted to finding alternatives to extract cellulose from byproducts of agricultural crops and/or textile wastes, both highly available at a very reduced raw material cost. Indeed, because of the ever-increasing consumption of cotton-based products, the amount of cotton waste generated, including pre-consumer (fiber linters, yarn slivers, fabric scraps from factory offcuts, unsold brand-new garments) and post-consumer (used and unwanted garments) wastes, has increased substantially in landfills [2,3]. In finished cotton fabrics, cellulose content can be up to 99% since non-cellulose components are eliminated during scouring and bleaching, which are routine preparation procedures. Considering the urgent demands for a circular economy and sustainable actions, researchers have been taking the first steps towards finding new and greener extraction systems for agricultural and textile wastes to endow the raw materials present within those wastes with a second life. This Special Issue brings together 10 original articles that detail the recent progress and new developments in this field.

Rizal et al., in a very compelling and critical overview of the worlds’ current situation regarding cotton waste fibers and their ineffective processing mechanism to mitigate their environmental impact, provided evidence of new work being conducted to employ these wastes in functional products. Indeed, different pre-treatment techniques were identified for their efficiency in extracting cellulose nanocrystals from cotton wastes, and many applications in the packaging and biomedical fields were highlighted [3]. Neto et al. explored and assessed the economic and environmental gains from the mechanical shredding of cellulose in cotton fabrics in a textile company, identifying the circularity associated with the adoption of such methods. Data suggested the existence of opportunities for the circular economy by resorting to mechanical recycling, even though there are still some limitations related to the consumption of electrical energy and amount of lubricants employed [4].

Nano-fibrillated cellulose extracted from waste peels of citrus sinensis by a chemical method involving alkali and acid hydrolysis and modified with silver nanoparticles also synthesized using extract of citrus sinensis skins as a reducing agent were engineered for heavy metal sorption. Data found these isolates and nanoparticles especially effective in removing cadmium and chromium particles from pharmaceutical effluent samples [5]. Rizal et al., demonstrated that cellulose nanofibrillated fibers isolated from waste cotton fabrics and combined with supercritical carbon dioxide via high-pressure homogenization could be used to enhance polylactic acid/chitin properties, particularly those related with the thermal-mechanical and wettability of the system [6]. Cellulose nanofibers have also been isolated from bleached carpet wastes using an innovative supercritical carbon dioxide treatment approach, which while isolating the materials also removed impurities. This treatment was unveiled as a green approach for enhancing the isolation yield of cellulose nanofibers from textile wastes with improved value and high quality [7]. Additionally, focusing on carpet fibers, Zughaibi et al., divulged the accuracy of a new analysis technique, the Direct Analysis in Real Time (DART™) coupled to an Accurate time-of-flight (AccuTOF™) mass spectrometer, for identifying distinct polymeric fibers in crime scenes, and highlighted its potential for forensic sciences [8].

Wang et al., engineered a flexible SERS substrate based on regenerated cellulose fibers, obtained from wastepaper, modified with gold nanoparticles through dry-jet wet spinning method, an approach turned eco-friendly by using ionic liquids. The gold nanoparticles were incorporated on the regenerated fibers through electrostatic interaction. The SERS scaffolding system was found very effective for identifying toxins and chemicals like dimetridazole in aqueous solutions [9]. Recycled newspaper fibers have also been collected and reinforced with high density polyethylene for potential outdoor applications. The composite samples were seen to improve water resistance and reduce loss of tensile strength in wet conditions, depending on the amount of stearic acid used to modify the newspaper fibers [10]. Additionally, Aghmashhadi et al., using waste banknote paper as raw matter, proposed the production of value-added products like bioethanol and biogas. In this study, the authors analyzed the influence of the presence and absence of ink on the fibers in the bioethanol and biogas yields. They determined that waste banknote paper without ink and treated with sulfuric acid and a nitrogen explosive decompression process could be a suitable feedstock for sustainable biorefinery approaches [11].

In the field of drug delivery, Al-Rajabi et al., explored a green synthesis method for developing a thermo-responsive cellulose hydrogel using cellulose extracted from oil palm empty fruit bunches. The thermo-responsiveness was guaranteed by the incorporation of Pluronic F127 polymer onto the hydrogels. A sustained release of silver sulfadiazine was observed over time. In the end, it was seen that the thermo-responsive cellulose-based hydrogel could give rise to cost-effective and sustainable drug delivery systems using abundantly available agricultural biomass [12].
